# Retraction: Extracellular regulated kinase 5 mediates osteoporosis through modulating viability and apoptosis of osteoblasts in ovariectomized rats

**DOI:** 10.1042/BSR20190432_RET

**Published:** 2026-02-04

**Authors:** Tuan-Mao Guo, Yan-Li Xing, Hai-Yun Zhu, Lan Yang, Guo-Xiong Liu, Xi-Min Qiao

**Keywords:** Extracellular regulated kinase-5, Osteoblast, Osteoporosis, Menopause, Calcium deposition, Bone mineral density

This article is being retracted from *Bioscience Reports* at the request of the Editor-in-Chief and the Editorial Board. This follows the receipt of a notification from a reader, alerting the Editorial Board to irregularities in the flow cytometry graphs, some of which suggest that the graphs could have been hand-drawn. The authors were contacted regarding the concerns and the retraction but have not yet responded or provided requested raw data. Given the extent of the issues raised, the Editorial Board stand by the decision to retract the article.

